# Sema3A secreted by sensory nerve induces bone formation under mechanical loads

**DOI:** 10.1038/s41368-023-00269-6

**Published:** 2024-01-19

**Authors:** Hongxiang Mei, Zhengzheng Li, Qinyi Lv, Xingjian Li, Yumeng Wu, Qingchen Feng, Zhishen Jiang, Yimei Zhou, Yule Zheng, Ziqi Gao, Jiawei Zhou, Chen Jiang, Shishu Huang, Juan Li

**Affiliations:** 1 State Key Laboratory of Oral Diseases & National Center for Stomatology & National Clinical Research Center for Oral Diseases & West China Hospital of Stomatology, Sichuan University, Chengdu, China; 2 Department of Orthopedic Surgery and Orthopedic Research Institute, West China Hospital, Sichuan University, Chengdu, China

**Keywords:** Bone, Stem-cell differentiation, Bone quality and biomechanics

## Abstract

Bone formation and deposition are initiated by sensory nerve infiltration in adaptive bone remodeling. Here, we focused on the role of Semaphorin 3A (Sema3A), expressed by sensory nerves, in mechanical loads-induced bone formation and nerve withdrawal using orthodontic tooth movement (OTM) model. Firstly, bone formation was activated after the 3rd day of OTM, coinciding with a decrease in sensory nerves and an increase in pain threshold. Sema3A, rather than nerve growth factor (NGF), highly expressed in both trigeminal ganglion and the axons of periodontal ligament following the 3rd day of OTM. Moreover, in vitro mechanical loads upregulated Sema3A in neurons instead of in human periodontal ligament cells (hPDLCs) within 24 hours. Furthermore, exogenous Sema3A restored the suppressed alveolar bone formation and the osteogenic differentiation of hPDLCs induced by mechanical overload. Mechanistically, Sema3A prevented overstretching of F-actin induced by mechanical overload through ROCK2 pathway, maintaining mitochondrial dynamics as mitochondrial fusion. Therefore, Sema3A exhibits dual therapeutic effects in mechanical loads-induced bone formation, both as a pain-sensitive analgesic and a positive regulator for bone formation.

## Introduction

The skeleton can sense mechanomechanical signals and convert them into biochemical signals, maintaining bone homeostasis.^1^ Bone is formed in high-strain areas and removed in low-strain areas, adapting bone morphology to the mechanical loads, termed strain adaptive bone remodeling.^2–4^ Orthodontic tooth movement (OTM) is a typical process of strain adaptive bone remodeling.^5^ The phenomenon of pain perception and regression following mechanical loads indicated that sensory nerves may be involved the bone remodeling process of OTM.^6,7^ Some observations suggested that crosstalk between bone and nerve affected the location and efficiency of adaptive bone remodeling.^8–11^ However, Heffner MA et al.^12^ implicated that the reduced peripheral sensory nerve function had little influence on the bone formation under mechanical loads. Consequently, the role of sensory nerves in stress-mediated bone formation awaits further validation.

Sensory nerve fibers are guided by physicochemical cues, in which nerve growth factor (NGF) and semaphorin 3A (Sema3A), as signal molecules for promoting axon extension and inhibiting growth cone, respectively, are co-located on the growth cone and axon.^13–16^ Besides their effects on nerve, Sema3A and NGF are also involved in bone formation. NGF is highly expressed in the ossification center, inducing innervation and bone formation.^17,18^ However, as the completion of osteogenic differentiation, osteoblasts highly express Sema3A, rather than NGF.^19^ These findings suggest that NGF and Sema3A play distinct roles in the initial stage and bone deposition stage, respectively, together determining the dynamic changes of bone formation and innervation. Therefore, exploring the sequential expression pattern of Sema3A and NGF may provide a new explanation for pain perception and bone formation during OTM.

Another aspect that requires clarification is the source of Sema3A, as Sema3A derived from different tissue appears to play important roles in different microenvironments. During skeletal development, nerve-derived Sema3A regulates sensory nerve development and indirectly influences bone remodeling.^20^ However, in aged bone, estrogen can promote osteocytes to secrete Sema3A and maintain bone homeostasis.^21^ And an another study suggested that deficiency of osteoblast-derived Sema3A led to osteopenia in long bones and lumbar spine.^22^ The determination of the specific tissue source of Sema3A during strain adaptive bone formation necessitates further investigation.

**Fig. 1:**
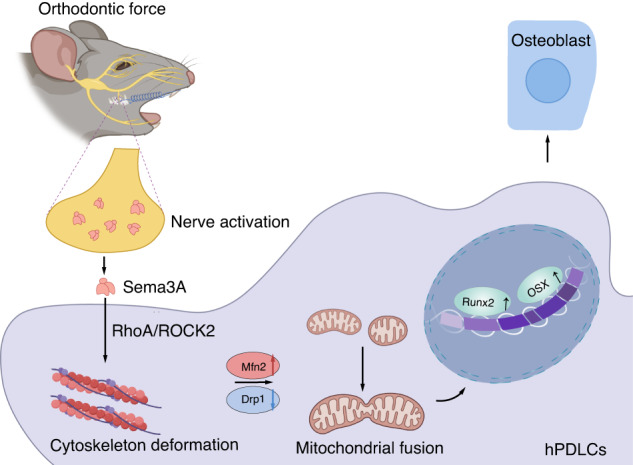
Schematic representation of Sema3A in mechanical loads-induced bone formation. In response to mechanical loading, the trigeminal ganglion is activated and releases Sema3A to interact with hPDLC. Sema3A further maintains cytoskeletal rearrangements in hPDLC by activating ROCK2 signaling, directing mitochondrial dynamics toward mitochondrial fusion. These responses are instrumental in preserving the osteogenic differentiation potential of hPDLC under mechanical loads

Here, we proposed that innervation was spatially and temporally correlated with alveolar bone formation during OTM. Sema3A, secreted by the trigeminal ganglion instead of periodontal tissue, played a crucial role in nerve withdrawal and bone formation under mechanical loads. Furthermore, Sema3A regulated cytoskeleton in human periodontal ligament cells (hPDLCs) under mechanical overload, leading to the restoration of mitochondrial fragmentation and subsequently inducing the osteogenic differentiation of hPDLCs (Fig. 1). These novel insights into the role of Sema3A in adaptive bone formation can offer new therapeutic avenues for bone diseases associated with mechanical overload, such as osteoarthritis and stress-induced fracture.

## Results

### Sensory nerves participate in mechanical loads-induced alveolar bone formation

The forces value suitable for mice OTM was investigated (Supplementary Fig. 1). The force of 5 g was sufficient to induce mesial movement of the first molar, and the distance of OTM increased with the increment of the applied force (Supplementary Fig. 1a). There was a reduction in bone mass on the tension side of the first molar when force of 50 g or higher was applied (Supplementary Fig. 1b–d). Adhering to the principles of light-force orthodontics, we employed 10 g to induce physiological OTM and 50 g to induce mechanical overloaded OTM.

**Fig. 2:**
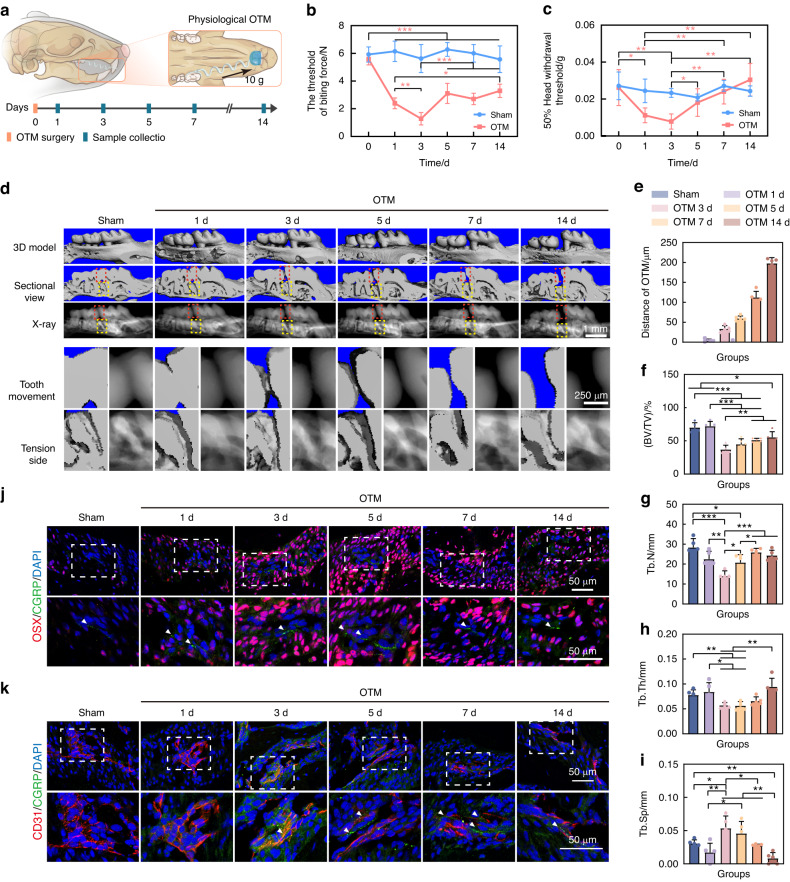
Spatial and temporal correlation of sensory nerves distribution and alveolar bone formation during OTM. **a** Schematic representation of 10 g induced physiological orthodontic tooth movement (OTM) and sample collection. **b**, **c** Bite force (**b**) and pain threshold in ear-temporal region (**c**) of mice gradually decreased before the 3rd day of OTM and increased afterward (*n* = 5. The red *p* value indicates the difference of the OTM group). **d** Micro-CT (μCT) reconstruction and X-ray showed the distance of OTM and the bone formation on the tension side of the first molar. The red and yellow boxes represent tooth movement and the distal alveolar bone of the first molar, respectively, and are shown below two rows for enlarged display. **e** Quantification of first molar moving distance (*n* = 5). **f**–**i** μCT quantification of BV/TV (**f**), Tb.N (**g**), Tb.Th (**h**), Tb.Sp (**i**) of the first molar distal alveolar bone during OTM (*n* = 5). **j**, **k** Immunofluorescence showed the spatial co-localization of CGRP^+^ sensory nerves with Osx^+^ osteoprogenitors (**j**) and CD31^+^ endothelial cells (**k**) in the periodontal tissues during OTM (white arrows indicate CGRP^+^ sensory nerves). All the quantitative data in Fig. 2 is presented as mean ± SD, and Two-tailed Student’s t-test was used for comparison. *P < 0.05; **P < 0.01; ***P < 0.001

The temporal distribution patterning of sensory nerves in alveolar bone was explored in 10 g induced physiological OTM (Fig. 2a and Supplementary Fig. 2a). A gradual decrease in bite force was observed during early OTM, reaching the lowest on the 3rd day. And the bite force increased after the 3rd day and remained stable after the 5th day of OTM (Fig. 2b). In addition, the 50% head withdrawal threshold displayed a similar trend, initially decreasing and subsequently increasing during OTM. Specifically, the lowest threshold value was also observed at the 3rd day (Fig. 2c). Correspondingly, calcitonin gene-related peptide (CGRP), a specific marker of sensory nerves, was found to gradually increase, peaking at the 3rd day after OTM (Supplementary Fig. 2b, c). Results above indicated that sensory nerves extended into the periodontal ligament following OTM, followed by a subsequent withdrawal starting on day 3 after OTM.

The temporal changes in tension-induced alveolar bone remodeling were next characterized. The first molar movement started on the 3rd day of OTM (Fig. 2d, e). And bone volume/total tissue volume (BV/TV), trabecular thickness (Tb.Th), and trabecular number (Tb.N) of alveolar bone in the tension side of first molar reached the lowest point on the 3rd day after OTM, followed by a gradual increase (Fig. 2d, f–i). Meanwhile, osteogenic marker Osx and endothelial cell marker CD31 were upregulated on the 3rd day, and maintained stable beyond the 7th day (Supplementary Fig. 2d–g). Furthermore, TRAP staining indicated heightened osteoclast activity preceding the 3rd day of OTM, gradually returning to normal levels thereafter (Supplementary Fig. 3). These findings suggested that the 3rd day of OTM marked a pivotal transition point in the alveolar bone remodeling, shifting from osteoclast-dominated pattern to osteogenic-dominated pattern.

The spatial correlation between sensory nerves and osteogenesis/angiogenesis during OTM was further examined. There was a noticeable enrichment of Osx^+^ osteoprogenitors and CD31^+^ endothelial cells around CGRP^+^ sensory nerves following the 3rd day of OTM (Fig. 2j, k). In sum, the temporospatial correlation between osteogenesis/angiogenesis and reinnervation suggested that sensory nerves may play an important role in mechanical loads-induced bone formation.

Capsaicin-induced sensory ablation was performed to confirm the important role of sensory nerves in mechanical loads-induced bone formation.^20^ The sensory ablation was confirmed by hot plate, Von Frey test, and immunofluorescence (Supplementary Fig. 4a–c). Capsaicin treatment itself had little discernible effect on alveolar bone. However, capsaicin significantly inhibited the bone formation in response to mechanical loads (Supplementary Fig. 4d–h). In addition, the expression of CD31 and Osx in the periodontal ligament of the tension side were also attenuated by capsaicin (Supplementary Fig. 4i–l). These results strengthened the critical role of sensory nerves in mechanical loads-induced alveolar bone formation.

### Mechanical loads induce Sema3A expression by sensory nerves

**Fig. 3:**
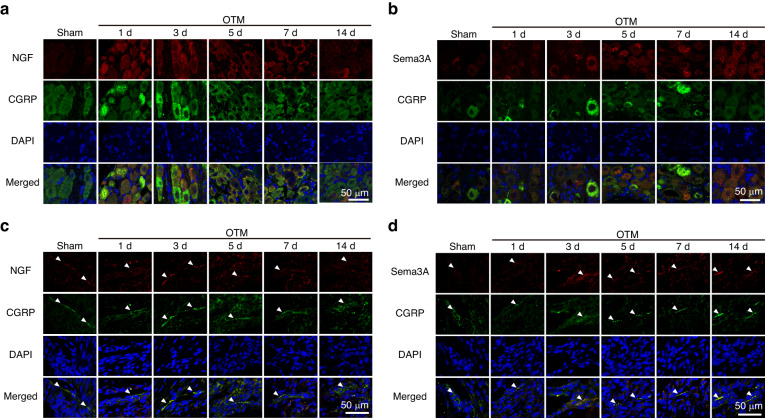
Sema3A from trigeminal ganglion serves as a key signal in late stage of OTM. **a**, **b** Immunofluorescence showed that NGF in the trigeminal ganglion increased rapidly before the 3rd day of OTM and then gradually decreased (**a**), while Sema3A in the trigeminal ganglion gradually increased after the 3rd day of OTM (**b**). **c**, **d** Immunofluorescence showed that NGF in the periodontal tissue increased rapidly before the 3rd day of OTM and then gradually decreased (**c**), while Sema3A in periodontal tissue increased gradually after the 3rd day of OTM (**d**). The co-localization with the CGRP^+^ nerve in periodontal tissue were indicated by white arrow

To elucidate the key factors for bone formation and nerve withdrawal in OTM, Sema3A and NGF expressed in vivo were assessed. Within the trigeminal ganglion, NGF increased on the 1st day of OTM and then decreased on the 3rd day, while a persistent increment in Sema3A expression was noted after the 3rd day of OTM (Fig. 3a, b and Supplementary Fig. 5a, b). The load-induced upregulation of Sema3A in trigeminal ganglion was further confirmed by western blot (Supplementary Fig. 5e, f). Same temporal expression patterns were discerned within the periodontal tissue, in which NGF demonstrated a rapid augmentation on the 1st day of OTM, followed by a gradual decline after the 3rd day, while Sema3A displayed an upsurge on the 3rd day after OTM (Fig. 3c, d and Supplementary Fig. 5c, d). It was worth noting that the highly expressed areas of Sema3A and NGF in periodontal tissue were always spatially colocalized with CGRP^+^ sensory nerves (Fig. 3c, d). Clinical samples indicated that orthodontic force also stimulated the expression of Sema3A in human periodontal tissue (Supplementary Fig. 6). To sum up, we found the continuously increased Sema3A and the progressively decreased NGF after the 3rd day of OTM.

**Fig. 4:**
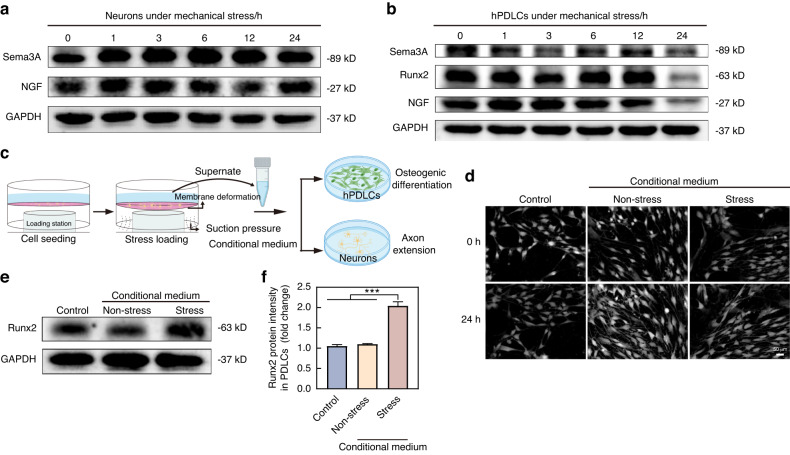
Mechanical loads promote neurons secret Sema3A. **a** Western Blot showed that mechanical loads promoted the expression of Sema3A in neurons. **b** Western Blot showed that 24-hour mechanical loads inhibited the expression of Sema3A, Runx2, and NGF in hPDLCs. **c** Schematic representation for exploring the efforts of the supernatant from neurons receiving 24-hour mechanical loads (conditional medium) on axon extension and hPDLCs differentiation. **d** 24-hour fixed-point photography showed that the conditional medium inhibited neurons growth. **e** Western Blot showed the conditional medium promoted the expression of Runx2 in hPDLCs. **f** Quantification of (**e**) (*n* = 3). mean ± SD, and Two-tailed Student’s t-test was used for comparison. ***P < 0.001

To confirm that neurons were the source of Sema3A, in vitro loading were performed on neurons and hPDLCs using a Flexcell system to apply a 24-hour 10% cyclical tension force. Before mechanical loads, we conducted identification of hPDLCs and mice trigeminal ganglion derived neurons. The results confirmed that the neurons were Tubulin Beta 3 Class III (TUBB3) positive and the hPDLCs were characterized as α-Smooth Muscle Actin (α-SMA) (+) and vimentin (+) cells but Cytokeratin 14 (CK14) (-) (Supplementary Figs. 7 and 8). In neurons, tension could continuously promote the expression of Sema3A over a 24-hour period (Fig. 4a and Supplementary Fig. 9a). Conversely, when hPDLCs were subjected to stretch for 24 hours, the expression of Sema3A, NGF, and the osteogenic differentiation-related factor Runx2 was found to be inhibited (Fig. 4b and Supplementary Fig. 9c–e). Additional investigations were conducted to explore the effect of neurons for regulating axon extension and the osteogenic differentiation of hPDLCs. Following exposure to 24-hour mechanical loads, the supernatant of neurons was collected as conditional medium to culture neurons and hPDLCs (Fig. 4c). We harnessed the precise positioning function of the high-content cell imaging system to investigate the impact of the conditional medium on axon extension. The conditional medium can impede the extension of neuron axons by comparing the dark-field images of neurons at the same location taken 24 hours apart (Fig. 4d). Further research showed that the conditional medium can promote the expression of Runx2 in hPDLCs (Fig. 4e, f). In conclusion, mechanical loads can induce neurons to release Sema3A, inhibiting axon sprouting and facilitating osteogenic differentiation of hPDLCs.

### Exogenous Sema3A restores the mechanical overload-suppressed bone formation

**Fig. 5:**
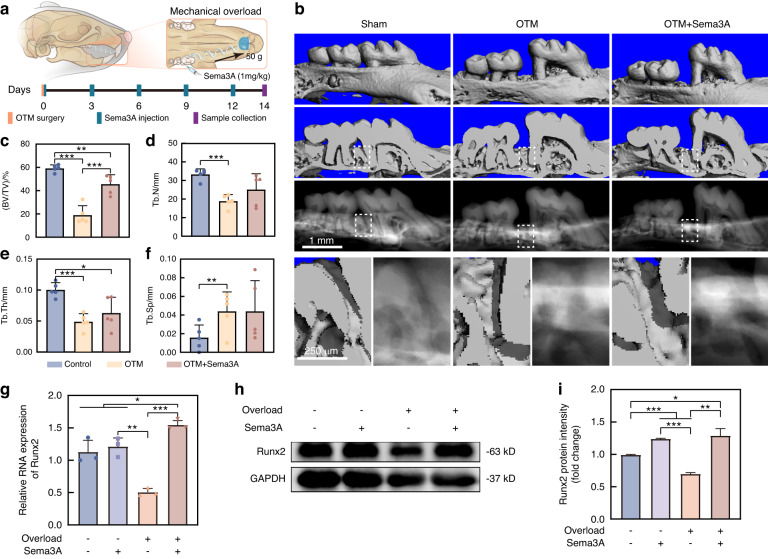
Exogenous Sema3A enhances mechanical overload-induced alveolar bone formation. **a** Schematic diagram of exogenous Sema3A injection into the mechanical overload (50 g) induced mice OTM. **b** μCT showed that exogenous Sema3A promoted mechanical loads-induced alveolar bone formation in the tension side of the first molar. **c**–**f** μCT quantification of BV/TV (**c**), Tb.N (**d**), Tb.Th (**e**), Tb.Sp (**f**) among Sham, OTM, and Sema3A treated OTM groups (*n* = 5). **g**, **h** exogenous Sema3A rescued the mechanical overload-induced decreased Runx2 RNA (**g**) and protein (**h**) expression in hPDLCs (*n* = 3). **i** Quantification of Runx2 protein in (**h**) (*n* = 3). All the quantitative data in Fig. 5 is presented as mean ± SD, and Two-tailed Student’s t-test was used for comparison. *P < 0.05; **P < 0.01; ***P < 0.001

To investigate the effect of Sema3A on enhancing strain adaptive bone formation, in vivo and in vitro experiments were conducted. The in vivo study used a mechanical overload (50 g)-induced OTM model (Fig. 5a and Supplementary Fig. 1). And the in vitro study performed the mechanical overload on hPDLCs to inhibit the osteogenic differentiation (10% stress, 0.5 Hz, 24 h, described in Fig. 4b).

The in vivo study was performed to validate the effects of Sema3A on mechanical overload-induced bone formation. Mechanical overload (50 g) was applied to the first molar of mice, and external Sema3A (1 mg /kg) was locally injected to the distal gingival sulcus of the first molar for every 3 days (Fig. 5a).^23^ Sema3A could promote the bone formation of the distal alveolar bone of the first molar in mice under mechanical overload, mainly as an increase in BV/TV (Fig. 5b, c). In addition, Osx^+^ osteoprogenitors in periodontal ligament increased following the addition of Sema3A, while CGRP^+^ sensory nerves surrounding periodontal ligament decreased (Supplementary Fig. 10).

As for the in vitro study, the concentration of Sema3A was first determined, and 100 ng mL^−1^ was used as the working concentration of Sema3A for the ability to promote proliferation and osteogenic differentiation of hPDLCs (Supplementary Fig. 11a–c). Further results confirmed that mechanical overload inhibited the ability of osteogenic differentiation in hPDLCs (Fig. 5g–i and Supplementary Fig. 12a–c). The exogenous Sema3A successfully mitigated these detrimental effects, leading to the upregulation of Runx2 and Osx expression and rescuing the osteogenic differentiation of hPDLCs (Fig. 5g–i and Supplementary Fig. 12a–c). These results suggested that Sema3A rescued the osteogenic differentiation of hPDLCs under mechanical overload.

### Sema3A prevents mechanical overload-induced F-actin hyperstretch

**Fig. 6:**
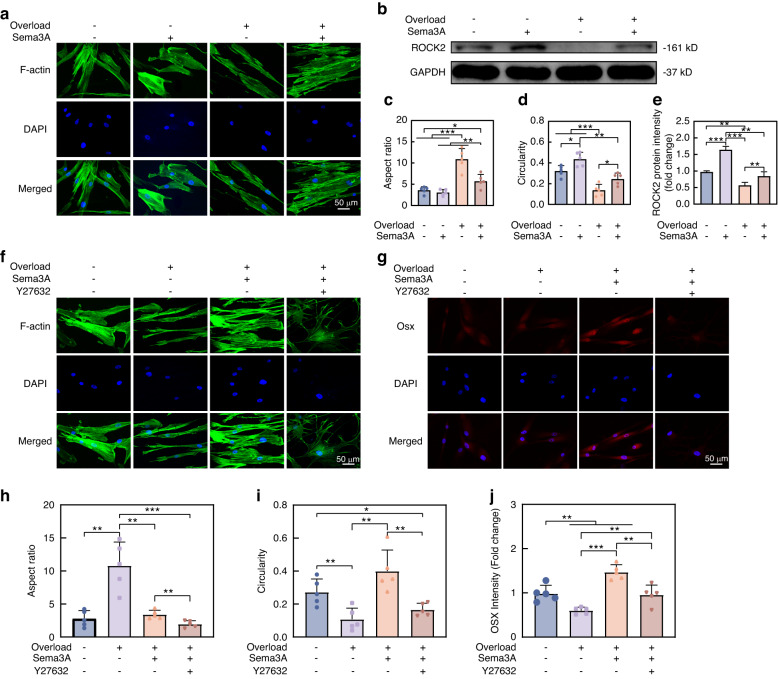
Sema3A maintains the spreading morphology of hPDLCs under mechanical overloads via the ROCK2. **a** Phalloidin staining showed that mechanical overload stretched hPDLCs, and Sema3A can maintain the spreading morphology of hPDLCs. **b** Western Blot showed that mechanical overload inhibited the expression of ROCK2 protein in hPDLCs, and exogenous Sema3A restored ROCK2 protein expression. **c**, **d** Quantification of the aspect ratio (**c**) and circularity (**d**) of hPDCLs in (**a**) (*n* = 5). **e** Quantification of ROCK2 protein in (**b**) (*n* = 3). **f** Phalloidin staining showed that the ROCK2 inhibitor Y27632 blocked the effect of Sema3A on F-actin spreading. **g** Immunofluorescence showed that Y27632 inhibited the efforts of Sema3A on the expression of Osx under mechanical overload. **h**, **i** Quantification of the aspect ratio (**h**) and circularity (**i**) of hPDLCs in (**f**) (*n* = 5). **j** Quantification of Osx in (**g**) (*n* = 5). All the quantitative data in Fig. 6 is presented as mean ± SD, and Two-tailed Student’s t-test was used for comparison. *P < 0.05; **P < 0.01; ***P < 0.001

Sema3A was proved to induce the axons retraction by regulating the cytoskeleton.^24^ Our observations also indicated that Sema3A reduced the aspect ratio and increased the roundness of hPDLCs (Supplementary Fig. 11c, e, f). Therefore, we hypothesized that Sema3A can regulate the F-actin of hPDLCs in mechanical microenvironment. Subsequent results revealed that 24-hour overload can realign the F-action of hPDLCs, characterized by the increased aspect ratio and reduced roundness. The administration of Sema3A decreased the aspect ratio of hPDLCs, making hPDLCs more spreading (Fig. 6a, c, d). The ROCK2 plays a significant role in cytoskeletal deformation and acts downstream of both Sema3A and mechanical loading.^25,26^ We confirmed that Sema3A can reactivate ROCK2 expression even under mechanical overload (Fig. 6b, e and Supplementary Fig. 13). Moreover, we utilized Y27632, an inhibitor for the RhoA/ROCK2 pathway, to validate the impact of the ROCK2 on Sema3A-induced osteogenic differentiation. We confirmed that Y27632 induced cytoskeletal disorder and diminished the expression of Osx and Runx2 in hPDLCs. Importantly, the introduction of Sema3A could not reverse the cytoskeletal collapse and osteogenic ability decline caused by Y27632, underscoring the pivotal role of the ROCK2 in the effects of Sema3A (Supplementary Fig. 14). Furthermore, when mechanical overloads applied, we still found that Y27632 not only disrupted the F-actin rearrangement mediated by Sema3A (Fig. 6f, h, i), but also inhibited Sema3A-stimulated Osx expression in hPDLCs (Fig. 6g, j), thereby negating the rescue effect of Sema3A on mechanical overload. Consequently, Sema3A mediates F-actin rearrangement of hPDLCs in mechanical microenvironment by regulating RhoA/ROCK2 pathway, thus affecting hPDLCs differentiation.

### Mitochondrial dynamics determine the response of hPDLCs to F-actin rearrangement

**Fig. 7:**
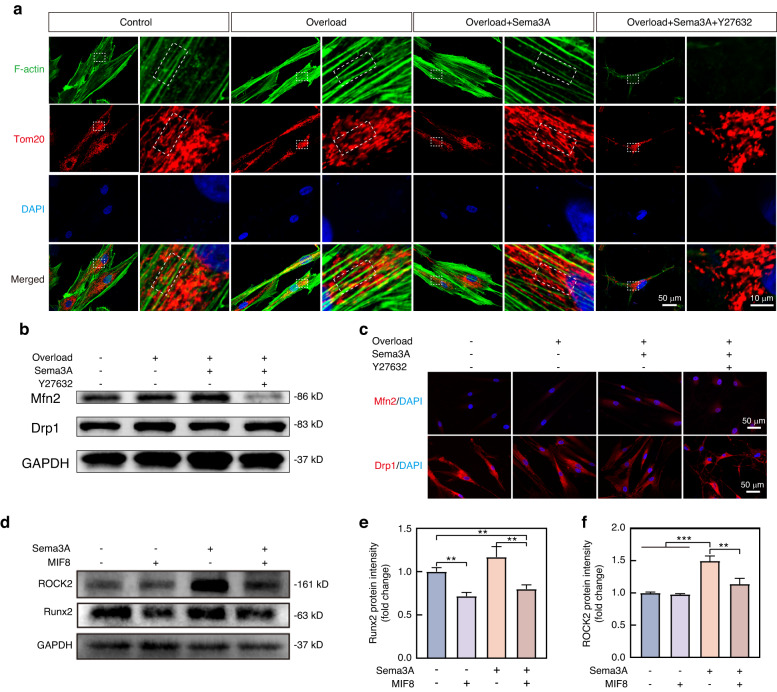
Sema3A promotes mitochondrial fusion in hPDCLs under mechanical overloads. **a** Tom20 and phalloidin staining demonstrated that mitochondria were distributed as a network along with F-actin in control group. Mechanical overload led to mitochondrial fragmentation and disassociation from F-actin. Sema3A treatment alleviated the mechanical overload-induced mitochondrial fragmentation, restoring the mitochondria network accompanied by F-actin. The ROCK2 inhibitor Y27632 disrupted the cytoskeleton, leading to the disintegration of the mitochondrial network in hPDLCs. **b** Western Blot revealed that Sema3A promoted the expression of Mfn2 protein in hPDLCs under mechanical overload, whereas the ROCK2 inhibitor Y27632 inhibits Mfn2 protein expression. **c** Immunofluorescence revealed that Sema3A restored the decreased Mfn2 expression and inhibited the Drp1 expression, and the restorative effect was attenuated by the ROCK2 inhibitor Y27632. **d** The Western Blot revealed that MIF8 exhibited little effects on the expression of ROCK2 protein, while effectively inhibit the expression of Runx2 protein in hPDLCs. Even in the presence of Sema3A, MFI8 retained the inhibitory effect on Runx2 expression. **e**, **f** Quantification of the Runx2 (**e**) and ROCK2 (**f**) protein in (**d**) mean ± SD, and Two-tailed Student’s t-test was used for comparison. *P < 0.05; **P < 0.01; ***P < 0.001

Mitochondria, as an organelle widely accompanying cytoskeleton, may serve as downstream signals of F-actin rearrangement.^27,28^ We used immunofluorescence of Tom20 and phalloidin staining to visualize the relationship of mitochondria and F-actin. Mitochondria in hPDLCs accompanied with F-actin and exhibited a networked structure in control group (Fig. 7a). The mechanical overload can lead to the scattered distribution of mitochondria among actin, mainly in fragments. Sema3A saved the mitochondria from fragmentation, redistributing mitochondria in a network (Fig. 7a). Importantly, Y27632 diminished the regulatory effect of Sema3A on mitochondria (Fig. 7a). Further studies were performed to figure out the mitochondrial dynamics during the processes. Mechanical overload suppressed the expression of Mitofusin-2 (Mfn2), a protein indicating mitochondrial fusion. Conversely, Sema3A reversed the mitochondrial dynamic by upregulating Mfn2 expression and downregulating dynamin-related protein 1 (Drp1), a protein associated with mitochondrial fission (Fig. 7b, c and Supplementary Fig. 15a–d). Y27632 interfered with the actions of Sema3A, thereby maintaining a fission-dominated mode of mitochondrial dynamics (Fig. 7b, c and Supplementary Fig. 15a–d).

We employed MFI8, a mitochondrial fusion inhibitor^29^ to confirm that mitochondrial fusion is a downstream for Sema3A-induced F-actin rearrangement. The 10 μmol/L MFI8 can induce mitochondrial fragmentation in hPDLCs (Supplementary Fig. 16a). Subsequent investigations demonstrated that MIF8 could inhibit the expression of Runx2 in hPDLCs. Importantly, the presence of Sema3A did not restore the inhibitory action of MFI8 on Runx2 expression (Fig. 7d–f and Supplementary Fig. 16b). The results established that Sema3A promoted osteogenic differentiation of hPDLSCs through mitochondrial fusion. Further investigations confirmed that MFI8 did not disrupt the cytoskeleton of hPDLCs, and it had little impact on the expression of the ROCK2 (Fig. 7d, f and Supplementary Fig. 16b). Conversely, Y27632-induced ROCK2 inhibition resulted in reduced Mfn2 expression and mitochondrial fragmentation (Fig. 7a, b). In summary, our findings supported that Sema3A promoted osteogenic differentiation of hPDLCs by upregulating ROCK2 under stress microenvironment, thereby stabilizing the cytoskeleton and then maintaining mitochondrial fusion.

## Discussion

This study found that in the tension-side of alveolar bone in OTM mice, osteogenesis and angiogenesis occurred after reinnervation and were spatially adjacent with the sensory nerves. Previous researches also observed a similar sequence of events, where innervation preceded vascular infiltration and bone deposition during bone development, fracture repair, and heterotopic ossification.^17,30,31^ Moreover, in clinical orthodontic treatment, pain perception was most evident in the early stage of force application, while bone deposition occurred in the later stage.^6^ This clinical phenomenon further suggests the potential role of sensory nerves in guiding bone formation. A recent study indicated that OTM can activate osteoclast process through sensory nerves.^7^ Our study further confirmed that sensory nerves act as sentinels to induce osteogenesis in mechanical loads-induced bone formation.

Another important question arises regarding the signals that mediate nerve withdrawal and osteogenesis under mechanical loading, as osteogenesis occur when pain subsides and sensory nerves exit. Many studies proposed that the NGF-TkrA signaling, as a forerunner, initiated nerve invasion and bone formation.^30,32,33^ However, the present study found that NGF expression increased at the initial stage in the trigeminal ganglion and periodontal tissue, but decreased later. Long et al.^34^ also observed a peak in NGF expression in the trigeminal ganglion on the 3rd day of OTM, followed by a decrease after the 5th day. Similarly, in clinical orthodontic treatment, noticeable pain and chewing weakness appeared at the initial stage, which subsided after 1–2 weeks.^35^ Aaron W James et al.^18^ also discovered that NGF expressed in skull defects decreased in the late stage of the repair process. This was consistent with the physiological phenomenon that sensory nerves were less distributed in mature bone tissue.^36^ Therefore, NGF may not be a key signal for loads-induced alveolar bone formation, especially in the late stages of alveolar bone formation.

When the expression of NGF decreases, it worth to figure out the signal that takes over its role to induces subsequent bone formation. Studies have indicated that after mesenchymal stem cells differentiated into osteoblasts, they highly expressed axon-repelling factors, such as Sema3A, Wnt4, and Shh, which discourage sensory nerve distribution, suggesting the crucial role of axon-repelling factors in osteogenesis.^19^ In diseases characterized by sensory sensitivity, such as atopic dermatitis, increased NGF level and decreased Sema3A level have been observed.^37^ Treatment can alleviate pain and reverse the abnormal expression pattern of Sema3A and NGF.^38,39^ Therefore, it is believed that Sema3A, a chemorepellent, plays an important role in pain extinction.^40^ Sema3A is also a key regulator of bone remodeling, promoting bone formation and inhibiting bone resorption.^20^ Furthermore, the present study found that Sema3A expressed in trigeminal ganglion remained high during OTM. And neurons highly expressed Sema3A instead of NGF under mechanical loads. Further in vivo and in vitro studies have confirmed that exogenous Sema3A can promote osteogenic differentiation of hPDLCs and induce bone formation even under mechanical overload. Therefore, Sema3A emerges as a key factor in bone formation in the late stages of OTM, mediating denervation and bone formation.

To clarify the source of Sema3A, the present study explored the expression of Sema3A in trigeminal ganglion and periodontal tissue. Previous studies have suggested that neurons-derived Sema3A is involved in bone development,^20^ while osteocytes-derived Sema3A is involved in maintaining bone homeostasis in aged mice.^21^ Another study indicated that deficiency of osteoblast-derived Sema3A led to osteopenia in long bones and lumbar spine.^22^ We observed increased expression of Sema3A in the trigeminal ganglion during OTM. In addition, Sema3A expressed in periodontal tissue is mainly co-located with CGRP^+^ sensory nerves. Furthermore, neurons, rather than hPDLCs, highly expressed Sema3A after the application of mechanical loads. These results further strengthen the significant role of neurons-derived Sema3A in loads-mediated bone formation.

This study also explored the downstream mechanism of Sema3A. Cytoskeleton deformation is required for the transformation from mechanomechanical signals to biochemical signals.^41,42^ Cyclic tensile stress can activate actin, making it distributing regularly and pointing to poles of cells.^43^ The present study further confirmed that hPDLCs became spindle-shaped under tensile loads, with actin arranged parallel to the tensile force. Sema3A, identified as a significant guidance cue, can induce F-actin to recombination, forming protrusions that guide the directional migration of dendritic cells.^44,45^ In addition, Sema3A can also lead to the loss of actin in neurons and induce the collapse of growth cones through RhoA/ROCK2 pathway.^13,24,46,47^ Our research further demonstrated that Sema3A upregulated ROCK2 protein in hPDLCs, resulting in a more spreading morphology.

Cytoskeleton deformation induced a series of intracellular cascade reaction. On the one hand, mechanical stress is transmitted to the nucleus through cytoskeleton - nucleoskeleton, which changed gene expression.^48^ On the other hand, intracellular organelles are also activated, leading to subsequent cellular biological reactions.^49^ Mitochondria, as a highly microenvironment-dependent organelle, are distributed along the cytoskeleton within cells.^27,28,50,51^ Actin is densely distributed in the constricted parts of mitochondria, and actin activation mediates the Drp1-dependent mitochondrial fission process.^27^ The present research also demonstrated that mitochondria transition from network to fragment due to the deformation of cytoskeleton caused by mechanical overload. Sema3A can maintain mitochondrial activity and guide neurite extension by regulating mitochondria.^52,53^ We further confirmed that Sema3A can stabilize the morphology of mitochondria and maintain the network structure in mechanical overloads.

As increased NGF expression and consequent innervation were the primary causes for pain in bone disease, anti-NGF antibody has garnered significant attention.^54^ Tanezumab, an anti-NGF antibody, was approved by FDA as a non-opioid analgesic in 2017. However, adverse bone reactions, including osteonecrosis, limit the clinical application of Tanezumab.^55^ The present study provided a novel therapeutic research direction for treating bone pain, namely recombinant human Sema3A. Such a treatment can not only relief pain perception, but also counteract the detrimental effects of stress on bone remodeling.^56^ These findings hold great significance for bone diseases, especially those caused by mechanical overloads.

## Materials and methods

### Ethics approval information

This study was complied with relevant ethical regulation for animal research, and was performed in strict accordance with the recommendations in the Guide for the Care and Use of Laboratory Animals of Sichuan University. Clinical sample collection and animal procedures were approved by the Ethics Committee of West China Hospital of Stomatology, Sichuan University (WCHSIRB-D-2022-086). All animal experiments followed the Animal Research: Reporting of In Vivo Experiments (ARRIVE) guidelines. All participants involved in this study signed an informed consent form prior to sample collection and were informed of the potential benefits and risks of participating.

### OTM model construction

All animals were maintained in a virus- and parasite-free barrier facility and exposed to a 12-hour/12-hour light/dark cycle under standard conditions in the Animal Center of Sichuan University, China. Six-week-old male C57/B6J mice (*n* = 30, 20–25 g) were purchased from Dossy (Chengdu, China). Thirty mice were randomly divided into an OTM group (*n* = 25) and a sham group (*n* = 5). The OTM model was established as described previously.^57^ 0.1 mm stainless wire was used to ligate NiTi coil springs (3 M Unitek, Monrovia, CA, United States) onto the cervix of the maxillary first molars and ipsilateral incisor. In physiological OTM, adjust the tightness of the ligation to generates ~10 g of force (measured by a vernier) to move the first molar, and fix the ligation wire into incisor (Fig. 2a). In mechanical overload-induced OTM, the 50 g of force was applied using a nickel-titanium tension spring for mesial movement of the first molar (Fig. 5a). The animal conditions and appliances were monitored daily during the study. Mice were tested for biting withdraws and Von frey test on 1st, 3rd, 5th, 7th, and 14th day after OTM.

### Capsaicin-induced sensory ablation

We used capsaicin to induce sensory nerve ablation as described previously.^20^ Before capsaicin injection, mice were deprived of drinking water for 6 h to prevent pulmonary edema. Capsaicin (Sigma, United States) is prepared with vehicle (Tween-80:ethanol:saline = 10:10:80 (v/v)). Six-week-old mice (*n* = 20, 20–25 g) were randomly divided into two groups, receiving two rounds of capsaicin (capsaicin group) or vehicle treatment (vehicle group). In each round, mice were daily injected subcutaneously above the dorsal spine for three consecutive days (10 mg/kg on day1 and day2, 15 mg/kg on day3). Two rounds of injections were separated by one week. The hot-plate experiment and von frey test were used to confirmed the sensory nerve ablation. And the mice in capsaicin/vehicle groups were then randomly divided into 10 g induced OTM group and Sham group.

### Von Frey test for paw and head

The 50% Paw withdrawal threshold (PWT) was measured by von Frey test as described previously.^58,59^ We used the modified Dixon up and down method to measure the mechanosensitivity of the paws. We selected 0.008, 0.02, 0.04, 0.07, 0.16, 0.32 g fiber filaments, using by gradient ascending method. Before the test, the mice were placed in an elevated chamber for 0.5 hour. Starting from the fiber with the minimum force, the fiber was applied perpendicular to the plantar surface of the hind paw for 2–3 seconds. Fiber with higher force was applied when the mouse exhibited little response. Fiber with low force was applied when the mouse exhibited a withdrawal response. After the first positive reaction was observed, the force value was recorded. And 5 other tests were applied, and the 5 force and reaction was also recorded (whether it’s negative or positive). The 50% PWT was determined by using the following formula: 50%PWT = 10[Xf+kδ]/10 000. Xf is the exact value (in log units) of the final test of von frey hair, K is the tabular value for the pattern of the last six positive/negative responses, and δ is the mean difference (in log units) between stimuli.

50% Head withdrawal thresholds (HWT) was measured as the PWT for mechanical pain thresholds of head and face.^60–62^ The fiber filaments were applied to ear-temporal region. Up and down method was also used to calculate 50% HWT.

### Bite force test

The bite-force test was used to provide objective evaluations of OTM-induced pain according to previous study.^63^ The mice were acclimated to the testing environment and trained to bite the biting sensor one day prior to bite force test. A bite-force measurement device (50.00 N ± 0.03 N; Nanjing Bio-inspired Intelligent Technology Co., Ltd., Nanjing, China) was used to measure the bite force, and the bite force was measured for three trials and the maximal bite force was regarded as the threshold of biting withdrawal.

### Hot plate test

A hot-plate instrument (PS-52, Sakura, Japan) was used for the thermosensitivity test of mice.^7^ The plate temperature maintained at (55 ± 0.5) °C. The mice were placed on the hot plate, and the latency time from all four claws touching the plate to the licking and shaking of the hind limbs was recorded. The test was repeated 3 times, and each test last for 40 s at most to avoid tissue damage. The mean of three latency time was used as the reaction time for each mice.

### Micro-CT

The mice were euthanized under excessive CO_2_ and the alveolar bone was collected. The alveolar bone was fixed in 4% paraformaldehyde for 24 hours and then washed with running water for 6 hours. Micro CT (SCANCO MEDICAL AG, Switzerland) was used for bone scanning. The isotropic resolution is 10 μm, and the X-ray and source potential was 70 kV with amperage of 200 μ.a. Three-dimensional reconstruction and histomorphometric analysis were carried out. The alveolar bone in the distal buccal root of the first molar was selected as the Region of interest (ROI) for tension-induced osteogenesis (200 μm × 300 μm × 200 μm).^64^ The structural parameters analysis included the bone volume/total tissue volume (BV/TV), trabecular thickness (Tb.Th), trabecular number (Tb.N), and trabecular separation (Tb. Sp) were calculated by the SCANCO software. The distance of OTM was measured as the closest distance from the most distal point of the first molar to the most mesial point of the second molar.^65^

### Immunofluorescence

Tissue samples were decalcified in 10 mM ethylene diamine tetraacetic acid (EDTA) for 3 weeks, then dehydrated overnight with 15% and 30% sucrose, respectively, and finally embedded with optimal cutting temperature compound (OCT) (Sakura, Japan). Tissue sections of 10-μm thickness were prepared with a Leica cryostat. Cell samples were fixed with 4% paraformaldehyde for 15 min, and then permeabilized with 0.3% Triton X-100 for 10 minutes and blocking with 3% BSA for 1 hour. Tissue sections or cells were incubated overnight with primary antibody at 4 °C. The primary antibodies used in this experiment are as follows: CD31(Abcam, ab182981, 1:200), CGRP (Abcam, ab36001, 1:200), Runx2 (Abcam, ab192256, 1:200), Sema3A (Abcam, ab23393, 1:200), Rock2 (Huabio, ER1706-48, 1:200), Drp1 (Abcam, ab184247, 1:200), and Mfn2 (Huabio, ER1802-23, 1:200). After washing the primary antibody, samples was incubated in the secondary antibodies for 2 hours. The secondary antibodies used are as follows: goat anti-rabbit Alexa Fluor 555 (Huanio, HA1123, 1:200), goat anti-rabbit Alexa Fluor 647 (Huabio, HA1123, 1:200), donkey anti-goat Alexa Fluor 555 (Abcam, ab15011). Immunofluorescence images were collected by a Nikon confocal microscope (Nikkon, N-Strom & A1, Japan).

### Western blot

The trigeminal ganglia from mice were first homogenized by a tissue homogenizer, and then samples were lysed by RIPA (Beyotime, China) containing 1% PMSF (Solarbio, China). The cells were directly lysed by RIPA with 1%PMSF. The proteins were boiled at 100 °C for 10 minutes. The proteins were isolated by 10% SDS-PAGE gel (Shanghai Epizyme Biomedical Technology Co., Ltd., China) and transferred to polyvinylidene fluoride (PVDF) membrane (Solarbio, China). After blocking with 5% skim milk, PVDF membrane was incubated with primary antibody overnight at 4 °C. The primary antibodies used in this experiment mainly include Runx2 (Abcam, ab192256, 1:1 000), Sema3A (Abcam, ab23393, 1:1 000), NGF (Huabio, ET1606-29, 1:1 000), Rock2 (Huabio, ER1706-48, 1:500), Drp1 (Abcam, ab184247, 1:1 000), Mfn2 (Huabio, ER1802-23, 1:1 000), and GAPDH (Huabio, ET1601-4, 1:2 000). HRP-conjugated secondary antibody (1:2 500, Huabio) was used to combined primary antibody. Super ECL Plus kit (US Everbright, China) was used to visualize proteins, and images was obtained by a Chemi Doc Touch Imaging System (Bio-RAD, USA).

### Clinical sample collection

Patient sample collection was performed with the approval of the Ethics Committee of West China Hospital of Stomatology, Sichuan University (WCHSIRB-D-2022-086). According to the clinical diagnosis, three patients who needed premolar extraction for orthodontic treatment were involved in the experiment. The inclusion criteria were as follows: 12–18 years old; no caries or restoration in premolars; no history of taking anti-inflammatory drugs during OTM. Informed consent of patients and their families was obtained before sample collection. In order to exert mechanical loads on premolars, we bonded bracket on the premolar in one side, but not on premolar on the other side (Supplementary Fig. 4a, b). The premolars were extracted at 1 month after the mechanical loads, and the RNA of periodontal tissue was extracted by trizol.

### hPDLCs culture

hPDLCs were extracted from premolars of patients aged 12–18 who needed tooth extraction for orthodontic treatment, and informed consent was obtained. Specifically, the middle third periodontal ligament of the premolar root was scraped off and then digested using type I collagenase (Biofroxx, Germany) at 37 °C for 0.5 hour. hPDLCs were cultured in α-MEM (Gibco, USA) containing 10% FBS (Bovogen, Australia) and 1% streptomycin‐penicillin (Gibco, USA) at 37 °C in a humidified atmosphere with 5% CO_2_. The medium was replaced every 3 days and hPDLCs were passaged when reached 70%–80% confluence using 0.25% Trypsin (Hyclone, USA). hPDLCs of passages 3–5 were used for further experiments.

### Trigeminal ganglia (TG) derived neurons culture

TG were harvested and TG-derived neurons were cultured following the description by Yamamoto et al.^66^ Mice were first received an injection of an overdose of sodium pentobarbital. The skull of mice was carefully exposed from the spinal cord end. Following brain remove, the TG became accessible. Once isolated, the TG was submerged in PBS with 10% penicillin and streptomycin. The ganglia were finely minced using microscopic scissors and then subjected to digestion with Accutase™ (Thermofisher, USA) for 1 hour. After centrifugation at 1 000 r/min for 3 minutes, the supernatant was aspirated, and the remaining tissue was resuspended in neuron culture medium and seeded in the culture plate pre-coated with 20 μg/mL laminin (Sigma, USA). The neuron culture medium was comprised of Nerobasel medium (Thermofisher, USA) supplemented with B27 (Thermofisher, USA) and L-glutamine (Thermofisher, USA).

### Application of mechanical loads on cells

For the in vitro experiment, hPDLCs and TG-derived neurons were seeded onto collagen І-coated six-well BioFlex® plates (Flexcell Int. Corp., Hillsborough, NC, USA). After the cells reached 70% to 80% confluence, the culture medium was replaced with α-MEM containing 1% FBS. Cells were then subjected to tensile strain with tensity of 10% and frequency of 0.5 Hz for different time (1 h, 3 h, 6 h, 12 h, 24 h) using a Flexcell® Tension Plus™ FX-5000™ system (Flexcell Int. Corp., Hillsborough, NC, USA). Cells plated on BioFlex® plates but not subjected to stretch served as controls.

### Exogenous Sema3A treatment for mechanical overload-induced OTM

Building upon the insights gleaned from literature and our findings (Supplementary Fig. 1), we applied the 50 g as mechanical overload force to induce mesial movement of the first molar in mice. In the Sema3A treatment group, we injected exogenous Sema3A at a dosage of 1 mg/kg into the distal periodontal tissue of the first molar immediately after establishing the OTM surgery. Subsequently, injections were repeated every three days for a duration of 14 days (Fig. 5a). After the treatment, we collected the maxilla for further tests.

### Statistical analysis

The experimental data were statistically analyzed using SPSS 21.0 software and GraphPad Prism 8.0. All data are presented as mean ± standard deviation (SD). The independent sample t-test was used for comparison between two groups, and analysis of variance was used for comparison among multiple groups. Statistical differences are expressed as *P < 0.05, **P < 0.01, ***P < 0.001.

### Supplementary information

Supplementary Materials
