# Supplementary material for: Sema3A secreted by sensory nerve induces bone formation under mechanical loads
Source: Int J Oral Sci. 2024 Jan 19;16:5. doi: 10.1038/s41368-023-00269-6 (PMC10796360; doi:10.1038/s41368-023-00269-6)
Supplement: Supplementary file 1 — Supplementary Materials [file 41368_2023_269_MOESM1_ESM.pdf]

Supplementary Materials for

Sema3A secreted by sensory nerve induce bone formation under mechanical loads

Hongxiang Mei[1], Zhengzheng Li[1], Qinyi Lv[1], Xingjian Li[1], Yumeng Wu[1], Qingchen Feng[1], Zhishen Jiang[1], Yimei Zhou[1], Yule Zheng[1], Ziqi Gao[1], Jiawei Zhou[1], Chen Jiang[1], Shishu Huang[2*], Juan Li[1*]

1. State Key Laboratory of Oral Diseases, National Center of Stomatology, National Clinical Research Center for Oral Diseases, West China Hospital of Stomatology, Sichuan University, Chengdu 610000, China
2. Department of Orthopedic Surgery and Orthopedic Research Institute, West China Hospital, Sichuan University, Chengdu, China.

Correspondence to: Shi-Shu Huang: h0794062@scu.edu.cn; Juan Li: lijuan@scu.edu.cn;

This PDF file includes:

Supplementary Fig.1 - 16

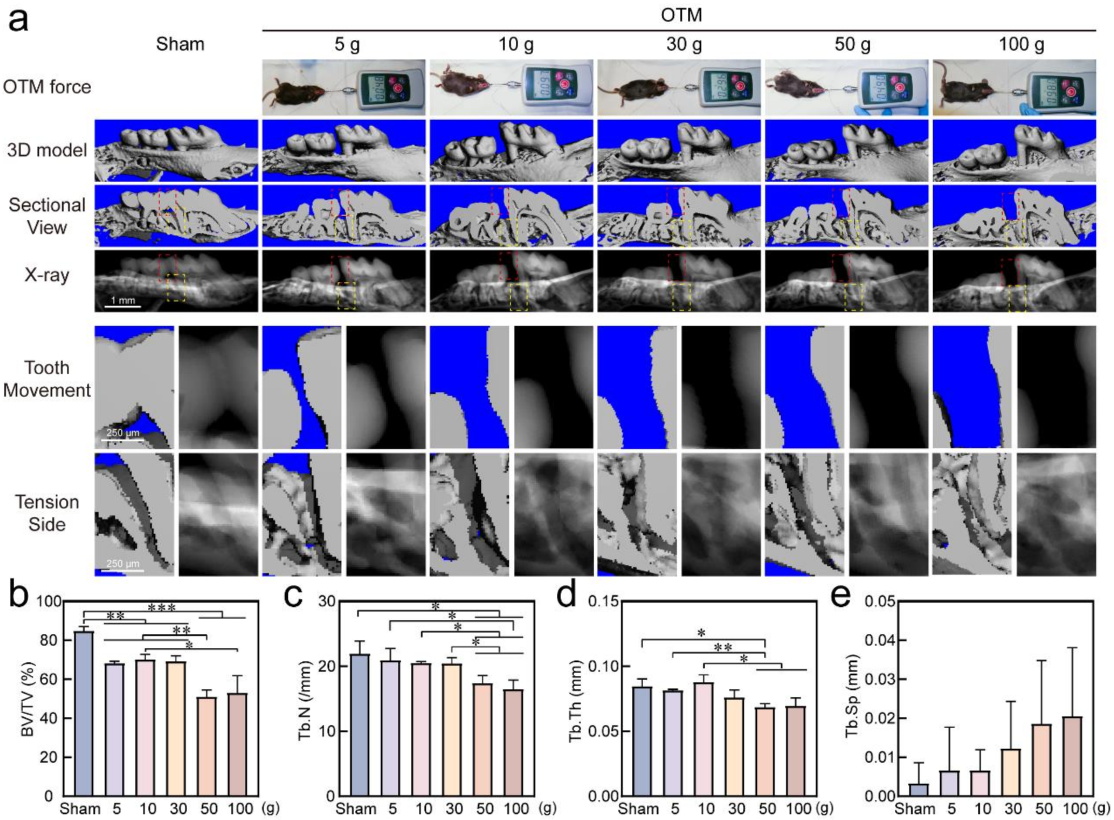

Supplementary Fig. 1. Effects of force value on the effectiveness of OTM and bone formation on the tension side in mice. a. Micro-CT (μCT) reconstruction and X-ray showed the distance of tooth movement and the alveolar bone formation in the tension side of the first molar induced by force of different values. The red and yellow dashed boxes represent tooth movement and the distal alveolar bone of the first molar, respectively, and are shown below two rows for enlarged display. **b-e.** μCT quantification of BV/TV (b), Tb.N (c), Tb.Th (d), Tb.Sp (e) of the first molar distal alveolar bone (n = 3).

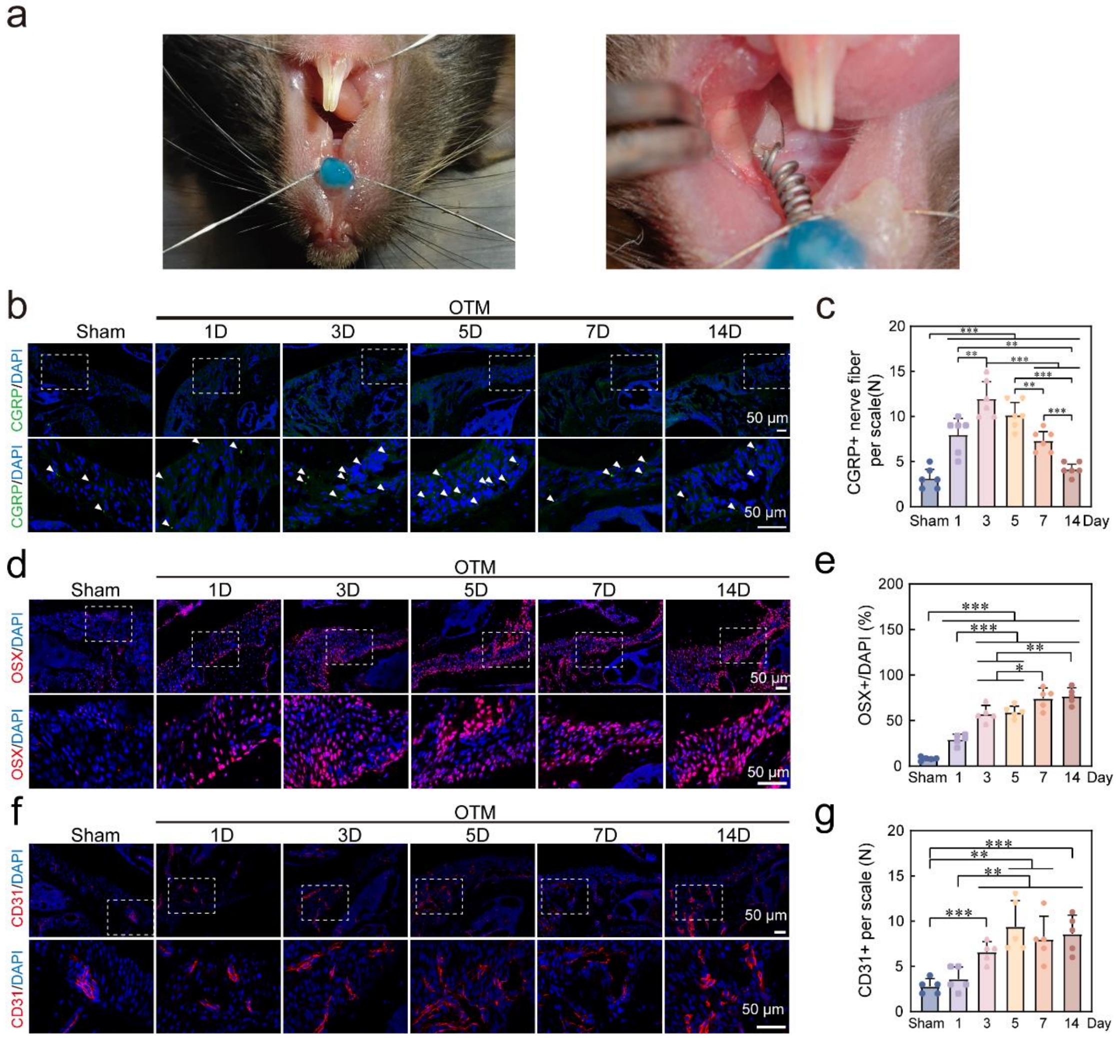

Supplementary Fig.2. The changes of CGRP⁺ sensory nerves, Osx⁺ osteoprogenitors and CD31⁺ endothelial cells during orthodontic tooth movement (OTM). a. Mice OTM model construction. **b, d, f.** Changes of $CGRP^+$ sensory nerves (b), Osx^+ osteoprogenitors (d), and $CD31^+$ endothelial cells (f) on the tension side of the first molar during OTM (white arrows indicate $CGRP^+$ sensory nerves). **c, e, g**. Quantification of $CGRP^+$ sensory nerves (c), Osx^+ osteoprogenitors (e), and $CD31^+$ endothelial cells (g) in the tension side of the first molar during OTM (n=5, mean ± SD, and Two-tailed Student's t-test was used for comparison. *P < 0.05; **P < 0.01; ***P < 0.001)

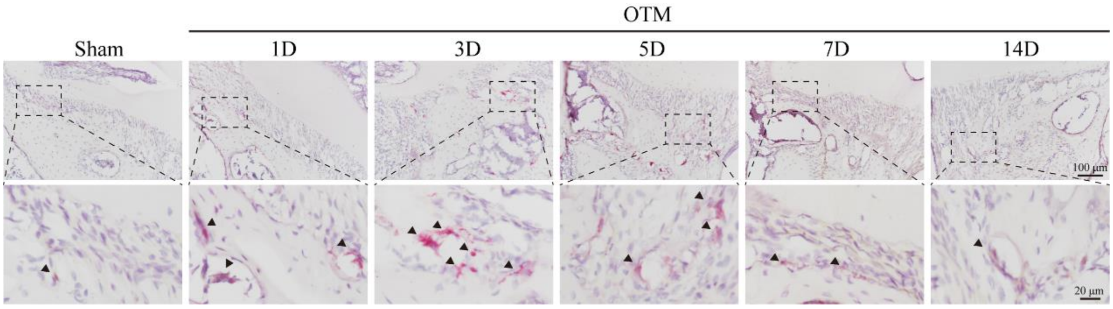

Supplementary Fig.3. The changes of osteoclast activity during OTM. TRAP staining of the alveolar bone on the tension side of the first molar during OTM.

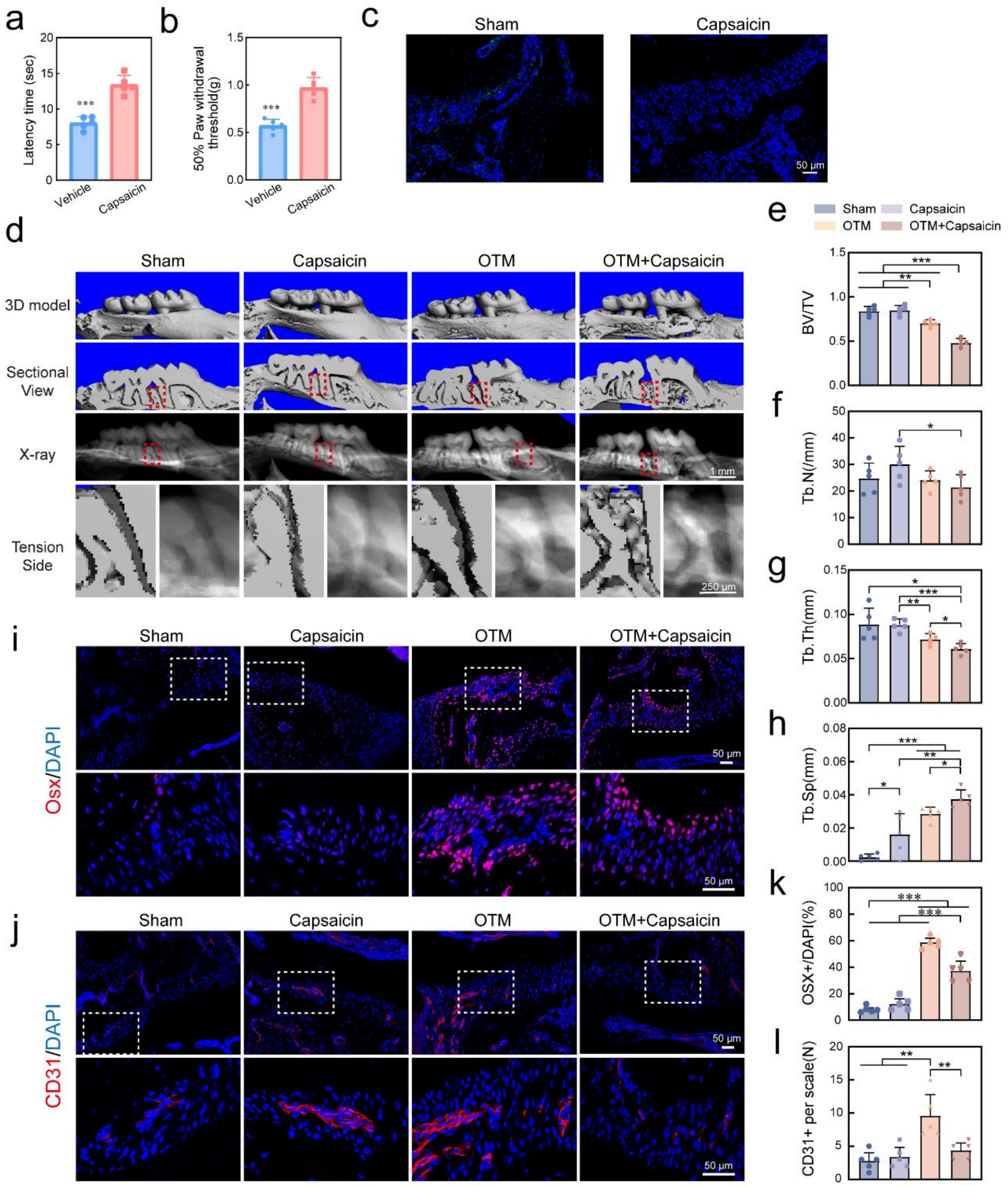

Supplementary Fig. 4. Sensory nerve ablation inhibits mechanical loads-induced alveolar bone formation. a. Hot plate test showed that capsaicin treatment prolonged the reaction time of mice to thermal stimulation (n=5). **b.** Von Frey test showed that capsaicin treatment prolonged the reaction time of mice to mechanical stimulation (n=5). **c.** Immunofluorescence showed that capsaicin treatment reduced the $CGRP^+$ sensory nerves on the tension side of the first molar of mice. **d.** μCT reconstruction showed that capsaicin treatment inhibited the alveolar bone formation on the tension side. **e-h.** μCT quantification of BV/TV (e), Tb.N (f), Tb.Th (g), Tb.Sp (h) of tension alveolar bone of the first molar among control, Capsaicin treated, OTM, and Capsaicin treated OTM

groups (n = 5). **i, j.** Immunofluorescence showed that capsaicin treatment reduced mechanical loads-induced upregulation of Osx^{+} osteoprogenitors (i) and $CD31^{+}$ endothelial cells in the tension side of the first molar (j). **j, l.** Quantification of Osx^{+} osteoprogenitor cells (j) and $CD31^{+}$ endothelial cells (l) in Supplementary Fig.4i and j. (All the quantitative data in Supplementary Fig.4 is presented as mean ± SD, and Two-tailed Student's t-test was used for comparison. $^{*}P < 0.05$; $^{**}P < 0.01$; $^{***}P < 0.001$)

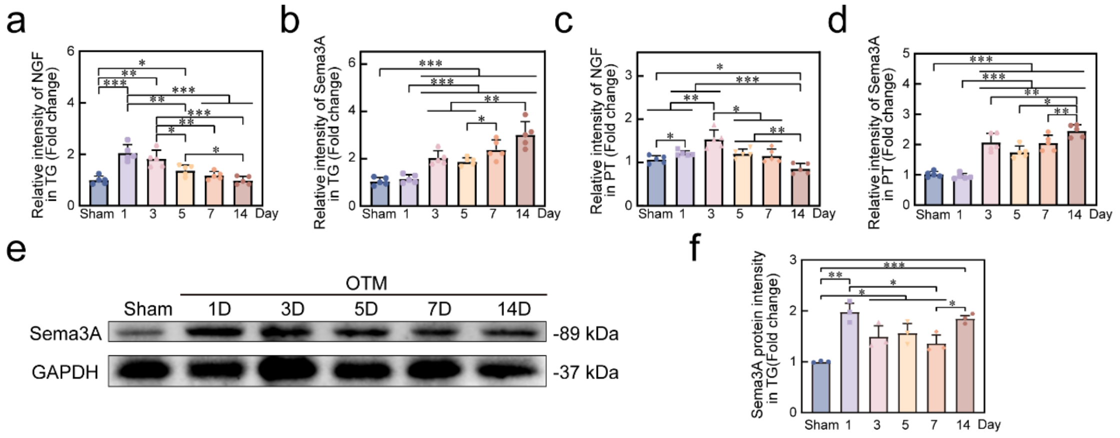

Supplementary Fig.5. Trigeminal ganglion highly expresses Sema3A protein during OTM. a-b. Quantification of NGF (a) and Sema3A (b) expressed in trigeminal ganglions (TG) in Fig. 3a-b (n = 5). **c-d.** Quantification of NGF (c) and Sema3A (d) in the periodontal tissue (PT) in Fig. 3 c-d (n = 5). **e**. Western Blot shows that OTM promoted the expression of Sema3A protein in trigeminal ganglion. **f**. Quantification of Sema3A protein in Supplementary Fig.5e (n=3). (All the quantitative data in Supplementary Fig.5 is presented as mean ± SD, and Two-tailed Student's t-test was used for comparison. $^{*}P < 0.05$; $^{**}P < 0.01$; $^{***}P < 0.001$).

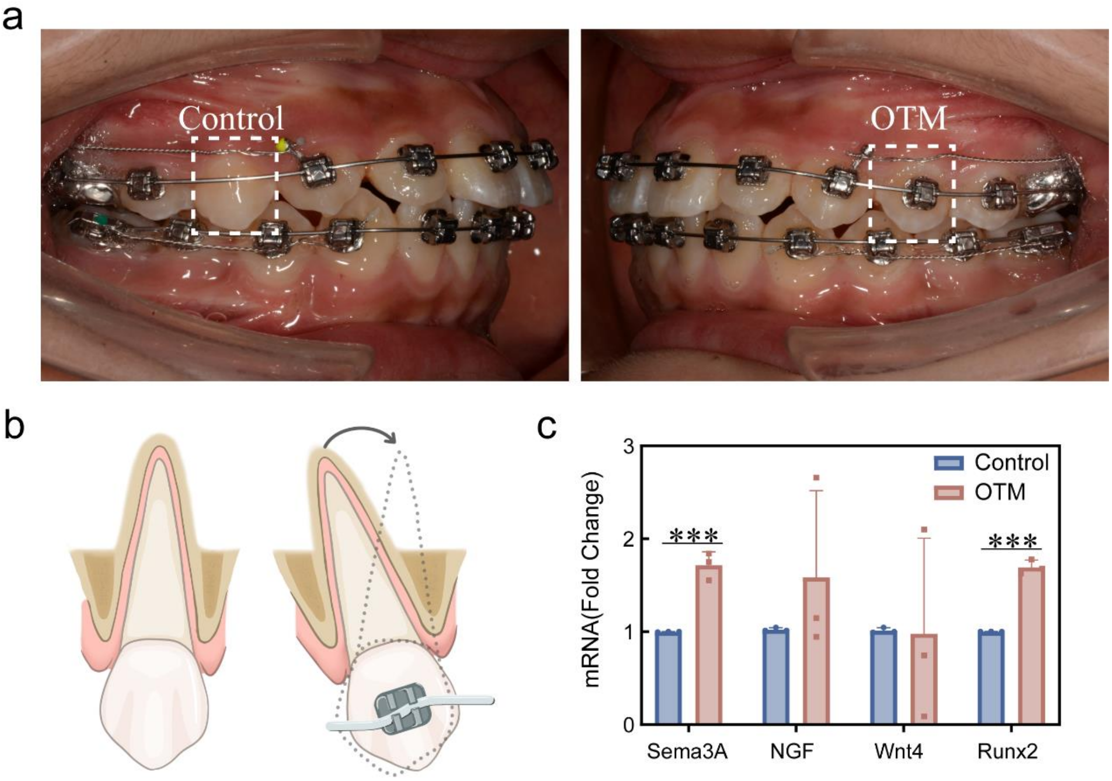

Supplementary Fig. 6. Clinical orthodontic treatment promotes the expression of Sema3A in human periodontal ligament. a. Clinical orthodontic force application for patients who needed premolar extraction for orthodontic treatment. **b.** Scheme of clinical force application on teeth and periodontal ligament. **c.** The efforts of clinical orthodontic force on the expression of Sema3A, NGF, Wnt4 and Runx2 in human periodontal ligament (n=3, data is presented as mean ± SD, and Two-tailed Student's t-test was used for comparison. ***$P < 0.001$)

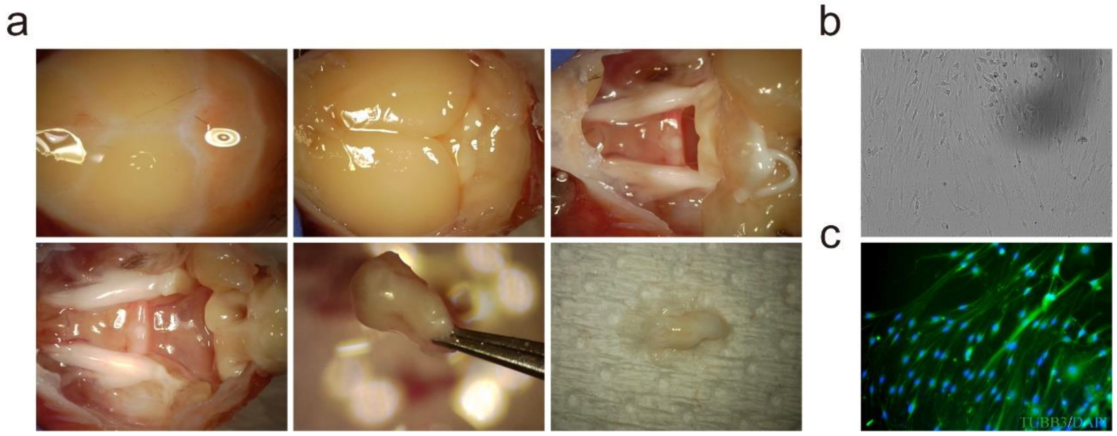

Supplementary Fig.7. Trigeminal ganglia anatomy and neurons culture. a. Anatomical process of the trigeminal ganglia. **b.** Morphology of trigeminal ganglia -derived neurons under microscope. **c.** TUBB3 immunofluorescence identification of trigeminal ganglia-derived neurons.

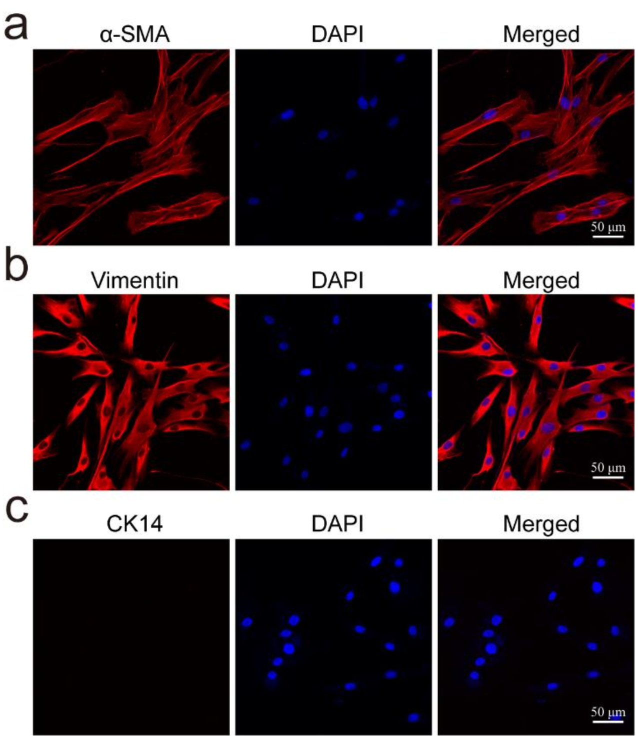

Supplementary Fig.8. Identification of human periodontal ligament cells (hPDLCs). a. α-Smooth Muscle Actin (α-SMA) immunofluorescence of hPDLCs. **b.** Vimentin immunofluorescence of hPDLCs. **c.** Cytokeratin 14 (CK14) immunofluorescence of hPDLCs.

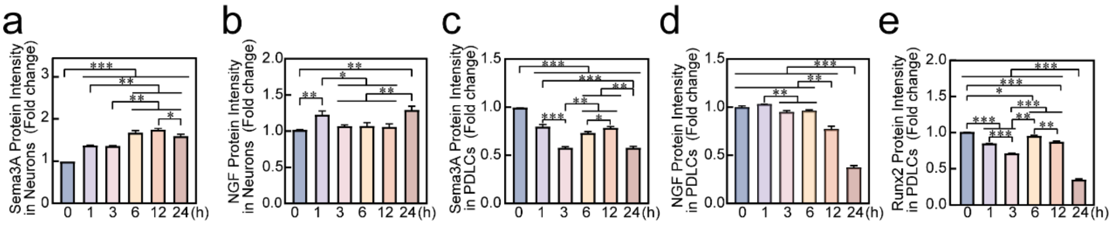

Supplementary Fig.9. a-b. Quantification of Sema3A (c) and NGF (d) expressed in neurons receiving mechanical loads in Fig. 4a (n=3). **c-e.** Quantification of Sema3A (e), NGF (f), and Runx2 (g) expressed in hPDLCs receiving mechanical loads in Fig. 4b (n=3). (All the quantitative data in Supplementary Fig.9 is presented as mean ± SD, and Two-tailed Student's t-test was used for comparison. $^*P < 0.05$; $^{**}P < 0.01$; $^{***}P < 0.001$).

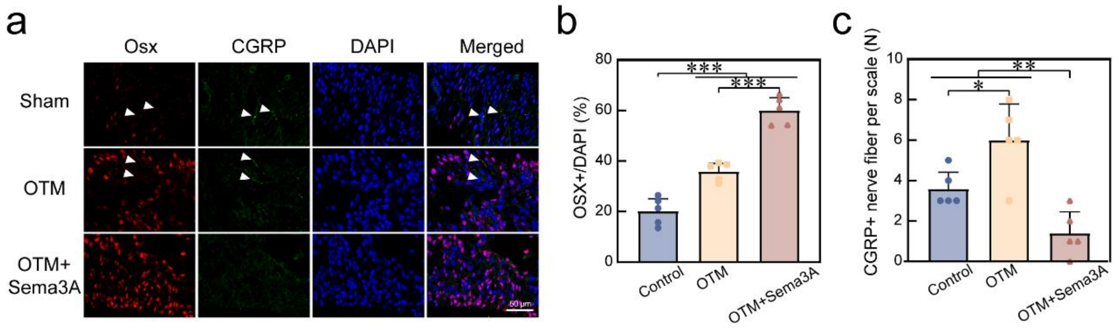

Supplementary Fig.10. Effects of exogenous Sema3A on osteogenic activity and sensory nerve distribution in the periodontal ligament on the tension side. a. Osx and CGRP immunofluorescent staining showed that Sema3A can increase Osx^+ osteoprogenitor cells and reduce CGRP+ sensory nerve fibers in the distal alveolar bone of the first molar (white arrow indicates the CGRP+ sensory nerve). **b-c.** Quantification of Osx+ osteoprogenitor cells (b) and $CGRP^+$ sensory nerves (c) in Supplementary Fig.10a (n = 5). (All the quantitative data in Supplementary Fig. 10 is presented as mean ± SD, and Two-tailed Student's t-test was used for comparison. *P < 0.05; **P < 0.01; ***P < 0.001).

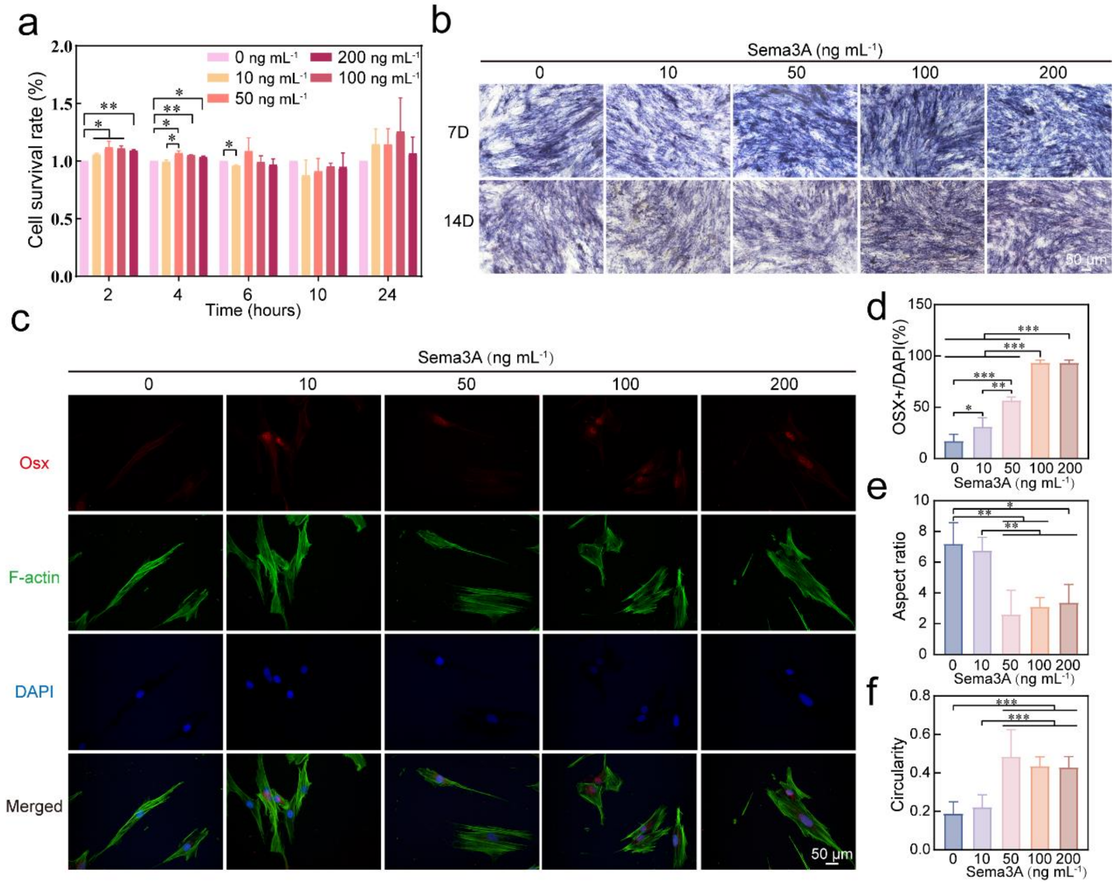

Supplementary Fig. 11. Effects of different concentrations of Sema3A on cell viability, osteogenic differentiation, and cell morphology of hPDLCs. a. CCK8 showed that Sema3A promoted cell proliferation within 4h (n=3). **b.** ALP staining showed that Sema3A at 100ng mL^{-1} had the strongest ability to promote hPDLCs osteogenic differentiation. **c.** Immunofluorescence showed that Sema3A promoted the Osx expression and cell spreading in hPDLCs. **d.** Quantification of Osx^{+} hPDLCs in Supplementary Fig. 11c（n=5）; **e-f.** Quantification of aspect ratio (e) and circularity (f) of hPDLCs in Supplementary Fig. 11c (n=5). (All the quantitative data in Supplementary Fig. 11 is presented as mean ± SD, and Two-tailed Student's t-test was used for comparison. *P < 0.05; **P < 0.01; ***P < 0.001).

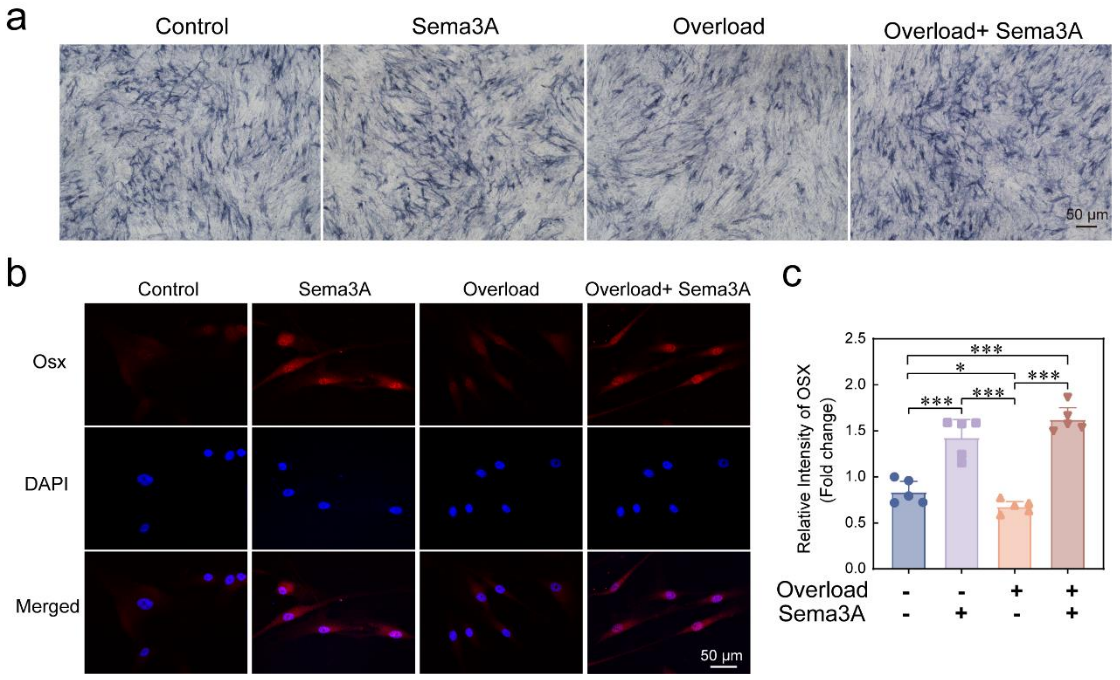

Supplementary Fig. 12. Sema3A restores mechanical overload-induced decreased osteogenic differentiation ability of hPDLCs. a. ALP staining showed that mechanical overload inhibited the expression of ALP in hPDLCs, and exogenous Sema3A restored ALP expression. **b.** Immunofluorescence showed that exogenous Sema3A promoted the expression of Osx in hPDLCs under mechanical overload. **c.** Quantification of Osx intensity in Supplementary Fig. 10b. (n=3, quantitative data is presented as mean ± SD, and Two-tailed Student's t-test was used for comparison. *P < 0.05; **P < 0.01; ***P < 0.001).

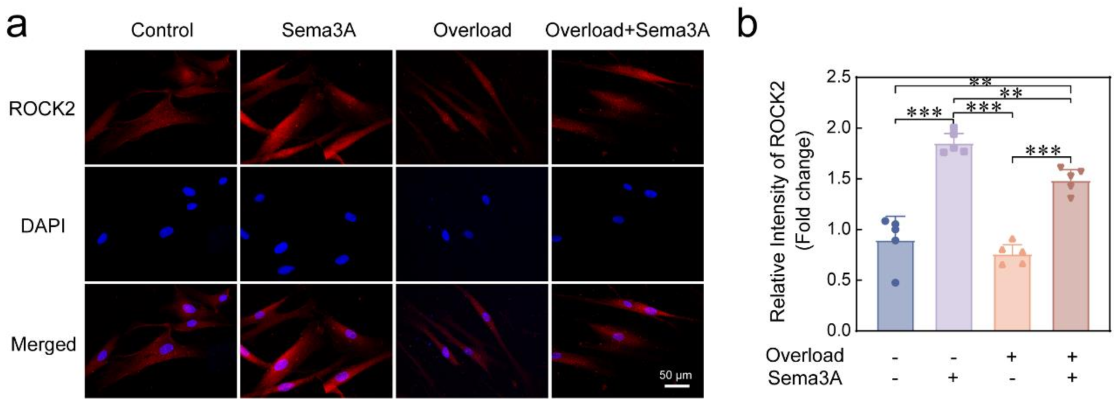

Supplementary Fig.13. Sema3A promotes the expression of ROCK2 protein in hPDLCs. a. Immunofluorescence showed that exogenous Sema3A promoted the expression of ROCK2 in hPDLCs under mechanical overloads. **b.** Quantification of the ROCK2 intensity in Supplementary Fig.13a (n=3, quantitative data is presented as mean ± SD, and Two-tailed Student's t-test was used for comparison. *P < 0.05; **P < 0.01; ***P < 0.001).

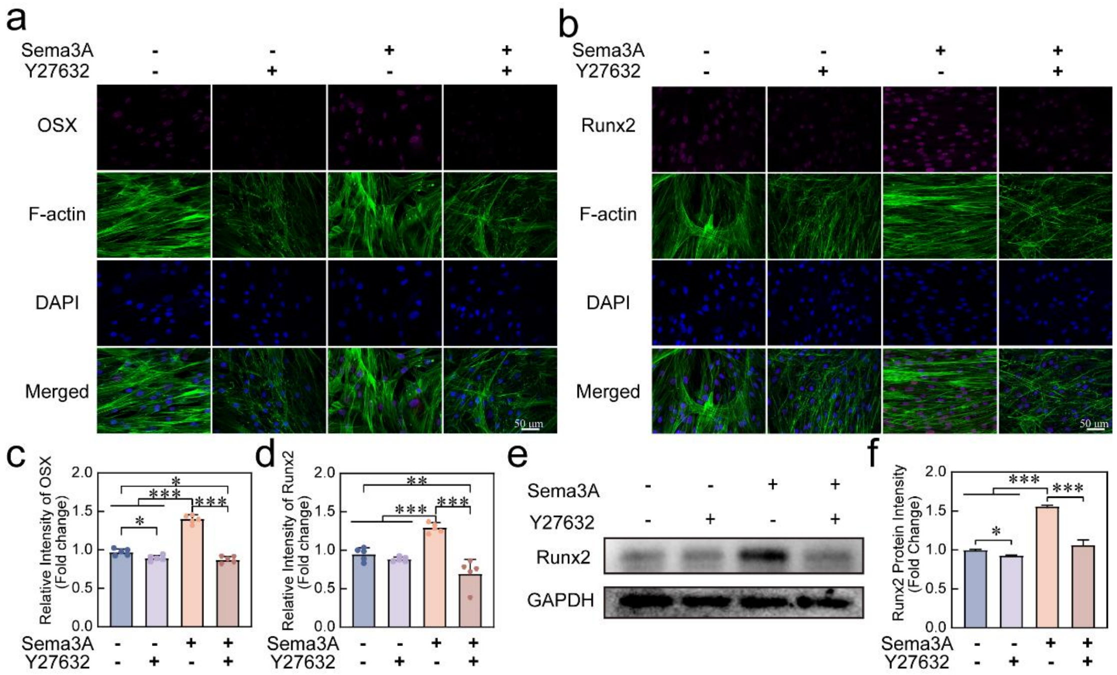

Supplementary Fig. 14. Y27632 eliminated Sema3A-mediated effects of osteogenic differentiation in hPDLCs. a-b. Immunofluorescence revealed that Y27632 treatment inhibited the expression of Osx (a) and Runx2 (b) in hPDLCs. And Y27632 effectively blocked the promotional effect of Sema3A on osteogenic differentiation. **c-d.** Quantitative analysis of fluorescence intensity in Supplementary Fig. 14. a (Osx, c) and b (Runx2, d). **e.** Western Blot demonstrated that Y27632 partially inhibited the expression of Runx2 protein in hPDLCs. Sema3A was unable to exert its positive effect on Runx2 expression of hPDLCs with the presence of Y27632. **f.** Quantitative analysis of Runx2 expression in Supplementary Fig. 14e. (All the quantitative data in Supplementary Fig.14 is presented as mean ± SD, and Two-tailed Student's t-test was used for comparison. *P < 0.05; **P < 0.01; ***P < 0.001).

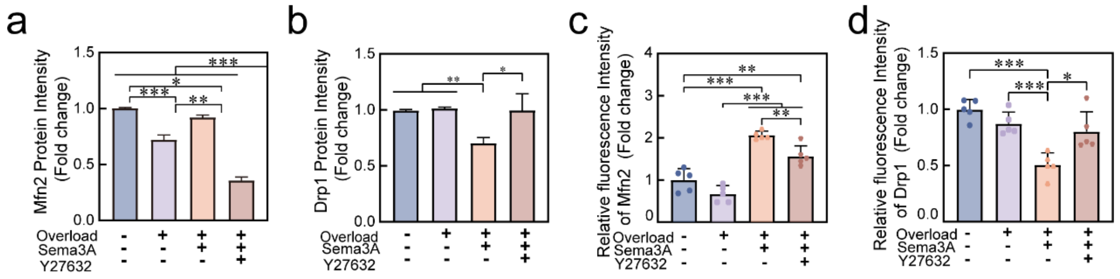

Supplementary Fig. 15. Quantification of data in Fig. 7. **a-b.** Quantification of Mfn2 (c) and Drp1 (d) protein in Fig. 7b. (n = 3). **c-d.** Quantification of the Mfn2 (c) and Drp1 (d) fluorescence intensity in Fig. 7c (n=5). (All the quantitative data in Supplementary Fig.15 is presented as mean ± SD, and Two-tailed Student's t-test was used for comparison. *P < 0.05; **P < 0.01; ***P < 0.001).

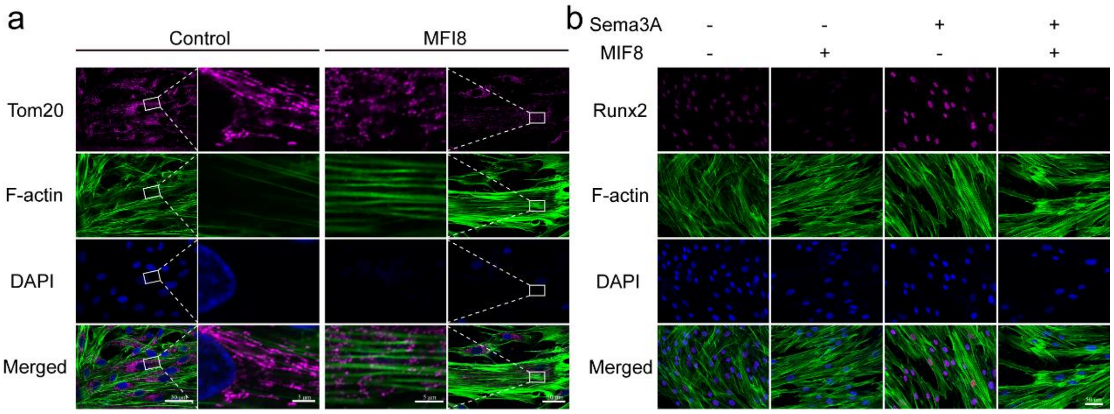

Supplementary Fig. 16. The inhibitory effects of MFI8 on mitochondrial fusion and Runx2 expression in hPDLCs. a. Tom20 staining illustrated that MFI8 effectively induced mitochondrial fragmentation. b. MFI8 inhibited the Sema3A-mediated increase in Runx2 expression.
